# Phase Transformations in MOFs Induced by Adsorbate Exchange

**DOI:** 10.1021/acs.langmuir.4c04626

**Published:** 2025-02-17

**Authors:** Alexander V. Neimark, Nicholas J. Corrente, François-Xavier Coudert

**Affiliations:** ! Department of Chemical and Biochemical Engineering, Rutgers, 242612 The State University of New Jersey, 98 Brett Road, Piscataway, New Jersey 08854, United States; @ Chimie Paris Tech, PSL University, CNRS, Institut de Recherche de Chimie Paris, 11 Pierre and Marie Curie, Paris 75231, France

## Abstract

Deformation of nanoporous materials induced by gas adsorption is a ubiquitous phenomenon that plays an important role in adsorption separations, gas and energy storage, nanosensors, actuators, secondary gas recovery, and carbon dioxide sequestration in coal and shale reservoirs. One of the most prominent examples is the breathing phase transformation in metal–organic frameworks (MOF) associated with significant volume variations upon adsorption and desorption of guest molecules. Here, we present a theoretical framework for the quantitative description of the breathing transitions upon adsorption of binary mixtures, drawing on the practically important example of the displacement of methane by carbon dioxide in the MIL-53 MOF. The proposed approach, which is based on the concept of adsorption stress, reveals the mechanisms of framework deformation and breathing phase transformation between the large pore (LP) and narrow pore (NP) conformations. We show that when pure CH_4_ adsorption proceeds entirely in the LP phase, even a small addition of CO_2_ makes the LP phase unstable and triggers conversion to the NP phase, and the reverse NP–LP transformation occurs upon further displacement of CH_4_ by CO_2_. The theoretical predictions of adsorption and strain isotherms are confirmed by an agreement with the literature experimental studies performed on MIL-53­(Al) at different CH_4_–CO_2_ mixture pressures and temperatures. The proposed general approach is applicable to other flexible nanoporous structures and gas mixtures.

## Graphical abstract

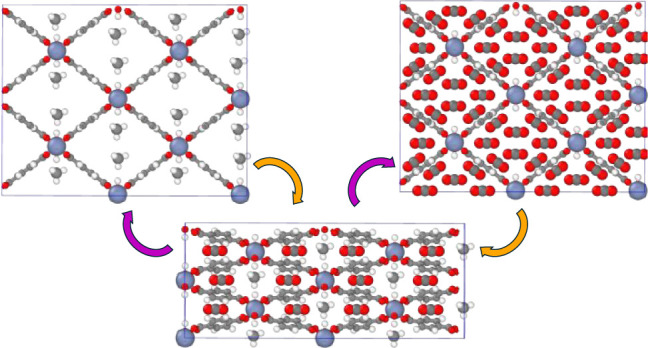

## Introduction

Phenomenon of phase transformations in flexible MOFs in the process of gas adsorption has been attracting continuing attention since the pioneering discoveries of the gate opening and breathing transitions,^1–3^ and more recently, the effect of negative gas adsorption.^4,5^ Numerous papers report potential applications of flexible MOFs for sensors, separations, gas and energy storage.^6–9^ The mechanisms of adsorption induced phase transformations were revealed in elaborated computational models based on ab initio, MD and MC simulations of various MOF systems.^10,11^ However, despite that most applications involve gas mixtures, the majority of experimental and theoretical works are restricted to studies of single component adsorption. Our objective is to build a theoretical model capable of predicting the breathing transitions triggered by adsorption of multicomponent gas mixtures, in particular in the process of adsorbate exchange, and to verify the proposed model on the experimental data on CH_4_ displacement by CO_2_ on MIL-53­(Al).^12^

MOFs of the MIL-53 family have a wine-rack framework with rhomboid pore channels, which may assume two characteristic narrow pore (NP) and large pore (LP) conformations.^13^ MIL-53 materials represent the case-study system for breathing transitions between NP and LP phases that are exhibited during adsorption–desorption cycles of various gaseous adsorbates, including CO_2_ and CH_4_, within certain ranges of temperatures and pressures.^14^ In the standard adsorption measurements, a vacuumed sample of MIL-53 in the LP phase is exposed to a gaseous adsorbate. As the gas pressure increases, two consecutive phase transitions may occur, first at a low pressure from LP to NP and then at a higher pressure from NP to LP, see Figure 1. The positions of breathing transitions depend on the gas pressure and temperature and are specific to the chemical nature of the adsorbate. In the case of multicomponent adsorption, the positions of transition depend on the composition of gas mixture.^12,15^

**Figure 1:**
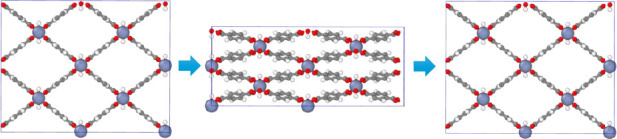
Schematic of LP–NP and NP–LP transitions in MIL-53.

Adsorption of CH_4_ and CO_2_ on flexible MOFs has been actively studied due to the acute practical importance of developing efficient adsorbents for natural gas purification and CO_2_ separation and capture.^16,17^ Boutin et al.^18^ studied breathing transitions on MIL-53­(Al) induced by single component adsorption of CO_2_ and CH_4_. It was shown that while adsorption of CO_2_ induces the consecutive LP-NP and NP-LP transitions in the wide range of temperatures, the breathing transitions during CH_4_ adsorption were found only at low temperatures below 250 K. At 273 K and higher temperatures, CH_4_ adsorption proceeds in the LP phase without the transition to the NP phase that is observed at lower temperatures. Later, Ortiz et al.^12^ showed on the same sample of MIL-53­(Al) that the displacement of CH_4_ by CO_2_ at fixed gas mixture pressure causes consecutive LP-NP and NP-LP transitions, similar to the single component adsorption of CO_2_.

The experimental isobar-isotherms of CO_2_–CH_4_ mixture given as functions of the CO_2_ fraction at fixed gas pressure and temperature demonstrate that the phase transformation between LP and NP phase depends on the mixture composition. While the LP-NP transition is not observed for pure CH_4_ at 273 K and higher temperatures, the displacement of CH_4_ by CO_2_ triggers the phase transformations, which are associated with the prominent steps on the adsorption isotherms. Addition of CO_2_ even in small amounts induces the LP-NP transition, and consecutive increase of the CO_2_ fraction leads to the NP-LP transition. The experimental adsorption–desorption isobars-isotherms exhibit a wide hysteresis loop associated with the NP-LP transition upon the increase of the CO_2_ fraction and the reverse LP-NP transition upon the decrease of the CO_2_ fraction. The hysteretic behavior is typical for breathing transitions; it shows that the transitions do not occur at the LP-NP phase equilibrium due to high nucleation energy barriers.^14^ The CO_2_ fractions at the onset of NP-LP and LP-NP transitions depend on the pressure and temperature in a nontrivial manner.

The conditions of NL-LP phase equilibrium is determined by the equality of the osmotic potentials in coexisting phases. Coudert^14^ suggested to use the ideal solution adsorption theory (IAST) to predict mixture coadsorption. This approach was applied in Ortiz et al.^12^ to calculate the osmotic potentials of NP and LP phases of MIL-53­(Al) upon adsorption of CO_2_-CH_4_ mixture and construct the NP-LP phase diagrams as functions of pressure, temperature, and composition. While the osmotic potential theory provides an approach for identifying the positions of equilibrium transitions, the problem of description of the experimentally observed hysteretic transitions affected by the energy barriers remains open.

In this work, we invoke the adsorption stress approach^19,20^ to account for the gas mixture effects on the breathing transitions. This approach was found to be efficient for analyses of breathing transition in MIL-53 upon single component adsorption.^11,13,18,19^ It was recently extended to the description of multicomponent adsorption and applied to predicting the deformation of coal upon competing CH_4_ and CO_2_ adsorption at ambient and geological conditions.^21^

## Methods

### Thermodynamics of Framework Deformation upon Adsorption of Gas Mixture

#### Osmotic Thermodynamic Potential and Adsorption Stress

To discuss thermodynamic properties of MOF crystals, it is instructive to present the thermodynamic potentials, adsorption isotherms, volume, and other extensive properties on a per unit cell basis. Equilibrium states of a flexible MOF crystal in the process of adsorption of a gas mixture at given composition (molar fractions {*y*_*i*_}), pressure *p*, and temperature *T* are determined by the osmotic thermodynamic potential of the unit cell of volume *v*:^22^

1 $$\omega_{\mathrm{os}}\left(\left\{\mu_{i}\right\},v,T\right)=f_{s}\left(v,T\right)+pv+\omega_{a}\left(\left\{\mu_{i}\right\},v,T\right)$$

Here, *f*_*s*_(*v*,*T*) is the free energy of the solid matrix and *ω*_*a*_({μ_*i*_},*v,T*) is the grand thermodynamic potential of the adsorbed phase controlled by the chemical potentials, {μ_*i*_}, of the gas mixture components. The latter depend on the mixture composition, pressure and temperature, μ*i* = μ_*i*_({*y*_*i*_},*p,T*).

In adsorption experiments, the effects of deformation are measured from the ideally vacuumed to negligibly small pressures, *p →* 0, reference nondeformed “dry” state of unit cell volume *v*_0_ without guest molecules present. Neglecting the deviatoric terms for a macroscopically isotropic solid, the framework free energy is related to the work of compression/expansion of the solid matrix from the reference unit cell volume *v*_0_ to current volume *v*,

2 $$f_{s}\left(v,T\right)=f_{s,0}+\int_{v_{0}}^{v}\sigma dv$$

Here, *f*_*s*,0_ = *f*_*s*_(*v*_0_,*T*) is the free energy of the “dry” solid matrix and σ is the volumetric stress,

$$\sigma ={\left(\frac{\partial f_{s}\left(V,T\right)}{\partial v}\right)}_{T}$$ 3

The grand thermodynamic potential of the adsorbed phase, *ω*_*a*_({μ_*i*_},*v,T*), commonly referred to in the adsorption literature as the integral work of adsorption, is related to the adsorption isotherms of mixture components, *N*_*i*_({μ_*i*_},*v,T*), according to the Gibbs equation,

$$d\omega_{a}{\left(\left\{\mu_{i}\right\},v,T\right)}_{v,T}=-\sum N_{i}\left(\left\{\mu_{i}\right\},v,T\right)d\mu_{i}$$ 4

The adsorption potential *ω*_*a*_({μ_*i*_},*v,T*) can be calculated by integrating the differential Gibbs equation (eq 4) along the trajectory of increasing pressure from the “dry” state at *p =* 0 to the given value *p* keeping the mixture composition {*y*_*i*_}, sample volume, and temperature fixed,

$$\omega_{a}\left(\left\{\mu_{i}\right\},v,T\right)=,-\int_{0}^{p}\left[\sum N_{i}^{\mathrm{theor}}\left(\left\{\mu_{i}\right\},v,T\right){\left(d\mu_{i}/dp\right)}_{\{y_{i}\},T}\right]dp$$ 5

Here, $N_{i}^{\mathrm{theor}}\left(\left\{\mu_{i}\right\},v,T\right)=N_{i}^{\mathrm{theor}}\left(\left\{y_{i}\right\},p,v,T\right)$ is the adsorption isotherm of component *i* in the mixture of composition {*y*_*i*_} at pressure *p* in the unit cell of volume *v* that is kept constant. The superscript “theor” distinguishes this theoretical isotherm from the experimental isotherm $N_{i}^{\exp}\left(\left\{\mu_{i}\right\},v_{0},T\right)$ that is measured on the evacuated sample characterized by a reference unit cell volume *v*_0_ It is worth noting that the experimental isotherm is recorded on a sample of varying volume and is affected by the adsorption-induced deformation effects. Moreover, $N_{i}^{theor}$ is the absolute adsorption isotherm that represents the total amount of adsorbed component *i*, while the experimentally measured isotherm $N_{i}^{exp}$ is commonly reported as the excess isotherm.^23^

The condition of mechanical equilibrium between the solid adsorbent and gas mixture stems from the minimization of ω_os_({μ_*i*_},*V,T*) with respect to the volume variation, at constant temperature *T* and chemical potentials of the mixture components {μ*i* = μ_*i*_({*y*_*i*_},*p,T*},

6 $${\left(\frac{\partial \omega_{\mathrm{os}}\left(\left\{\mu_{i}\right\},v,T\right)}{\partial v}\right)}_{\left\{\mu_{i}\right\},T}=0;,\Rightarrow \sigma =\sigma_{a}-p$$

Here, σ_*a*_ is the adsorption stress exerted by the guest molecules, which is rigorously defined as the negative derivative of the grand thermodynamic potential of the adsorbed phase *ω*_*a*_({μ_*i*_},*v,T*) with respect to the unit cell volume *v,* ^21,23^

$$\sigma_{a}=-{\left(\frac{\partial \omega_{a}}{\partial v}\right)}_{\{\mu_{i}\},T}$$ 7

The matrix deformation upon adsorption is determined by the adsorption stress, σ_a_. In the simplest linear elastic approximation, the volumetric strain, ε, of a macroscopically isotropic solid is defined by the difference between the adsorption stress and the external pressure *p* through the volumetric elastic modulus (or bulk modulus) of the solid, *k*, as^19,20^

8 $$\varepsilon =\frac{v-v_{0}}{v_{0}}=\sigma /k=\left(\sigma_{a}-p\right)/k$$

Here, the strain and the adsorption stress are defined with respect to the reference state of the undeformed “dry” sample of unit cell volume *v*_0_ evacuated at negligibly small pressures, *p* → 0, σ_*a*_ *→* 0.

The concept of the adsorption stress links the adsorption thermodynamics with the poromechanics^24,25^ allowing for predicting the adsorbent deformation in the process of gas adsorption. To this end, one has to determine the dependence of the theoretical adsorption isotherm $N_{i}^{theor}\left(\left\{\mu_{i}\right\},v,T\right)$ in the unit cell of volume *v* that can be done by using various molecular simulation and theoretical methods, using the direct Monte Carlo simulations,^26,27^ density functional theory,^28–32^ or conventional adsorption models parametrized for a given system.^18,19,21,33,34^

#### Equilibrium between NP and LP Phases

Experimental adsorption and desorption isotherms in MIL-53 materials always form a prominent hysteresis loop with characteristic steps at the NP-LP (or LP-NP) transition during adsorption and the reverse LP-NP (or NP-LP) transition during desorption. The conditions of NP-LP phase equilibrium are achieved at a certain equilibrium pressure inside the hysteresis loop. The adsorption–desorption hysteresis indicates that the equilibrium NP and LP states are separated by a large nucleation barrier that cannot be overcome in the experiments with a finite observation time. As the gas pressure increases (or decreases) beyond the equilibrium pressure, the system passes through a series of metastable NP (or LP) configurations upon reaching the *threshold stress*, $\sigma_{t}^{NP}$ (or $\sigma_{t}^{LP}$ ), at which the elastically deforming solid matrix becomes unstable, causing plastic deformation and transition to a stable LP (or NP) phase.^19^

The true thermodynamic equilibrium between coexisting within one crystal NP and LP phases implies the equality of the osmotic potentials in coexisting NP and LP unit cells,

9 $$\omega_{\mathrm{os}}^{NP}\left(\left\{y_{i}\right\},p_{e},v_{e}^{NP},T\right)=\omega_{\mathrm{os}}^{LP}\left(\left\{y_{i}\right\},p_{e},v_{e}^{LP},T\right)$$

Here, $\omega_{os}^{NP}$ and $\omega_{os}^{LP}$ are defined by the general eqs 1 and 2 applied respectively to the NP and LP phases. Using the linear stress–strain relationship (eq 8), the equilibrium condition (eq 9) is presented, as

$$f_{s,0}^{NP}+v_{0}^{NP}\frac{{\left(\sigma_{a,e}^{NP}-p_{e}\right)}^{2}}{2k^{NP}}+p_{e}v_{e}^{NP}+\omega_{a}^{NP}\left(\left\{y_{i}\right\},p_{e},v_{e}^{NP},T\right),=f_{s,0}^{LP}+v_{0}^{LP}\frac{{\left(\sigma_{a,e}^{LP}-p_{e}\right)}^{2}}{2k^{LP}}+p_{e}v_{e}^{LP}+\omega_{a}^{LP}(\left\{y_{i}\right\},p_{e},v_{e}^{LP},T)$$ 10

Subscript “e” here and below denotes the values of pressure and unit cell volumes at the NP-LP (or LP-NP) phase equilibrium.

Equation 10 contains three structural parameters, in addition to the parameters of the adsorption isotherms in the NP and LP phases, which are needed in order to determine the position of NP-LP phase equilibrium: the difference of the free energies of empty NP and LP phases, $\Delta f_{s,0}=f_{s,0}^{NP}-f_{s,0}^{LP}$ , and the volumetric moduli, *k^NP^* and *k* ^LP^. These parameters can be calculated from quantum mechanical minimization of crystal structures of ideal empty NP and LP phases.^11^ Alternatively, the volumetric moduli can be estimated from the experiments on mechanical compression.^21^ In the following analysis we adopt the reported values of *k* ^*NP*^ = 10 GPa and *k* ^*LP*^ = 2 GPa for MIL-53­(Al).^13^

It should be noted that the breathing transitions are characterized by a significant hysteresis between the adsorption and desorption isotherms so that the adsorption process proceeds through the metastable states in NP and LP phases. The true thermodynamic equilibrium between NP and LP phases is not experimentally observed, yet it can be predicted theoretically and provides additional information for a proper parametrization of the model, as shown below.

#### Langmuir Model of Binary Adsorption

To calculate the adsorption stress, it is necessary either to invoke a particular theoretical model for the mixture adsorption isotherm, like the commonly used Langmuir or Dubinin models,^21,34^ or to construct a series of isotherms in the MOF structures of varying cell volume.^11^ Here, we apply the conventional Langmuir model of binary adsorption,

11 $$N_{i}=\frac{N_{i}^{0}K_{i}y_{i}p}{1+K_{1}y_{1}p+K_{2}y_{2}p}$$

where $N_{i}^{0}$ and *K*_*i*_ are, respectively, the maximum adsorption capacity and the Langmuir parameter of individual components, *i* = 1,2. The Langmuir model is a commonly used model for description of adsorption on microporous adsorbents, including adsorption of CH_4_ and CO_2_ on MOFs.^14,19^ Being applied to 3D structures of MOFs, the Langmuir equation (eq 11) is considered as a practical empirical model; it has apparent limitations and implies the ideal gas mixture. The practical advantage of the Langmuir model is that it depends on the pairs of parameters, $N_{i}^{0}$ and *K*_*i*_, which characterize adsorption of the individual components and have a clear physical meaning. These parameters vary upon the alteration of the sample volume affected by deformation. The Langmuir parameter, *K*_*i*_, depend on the energy of adsorption and temperature via the van’t Hoff equation,

12 $$K_{i}\left(T\right)∼exp\left[-\frac{\Delta H_{i}}{RT}\right]$$

where Δ*H*_*i*_ is the equilibrium enthalpy of adsorption of species *i*.^35^

The binary Langmuir model (eq 11) allows for a direct derivation of the grand thermodynamic potential of the adsorbed phase, *ω*_*a*_({μ_*i*_},*V,T*), by integration of eq 4 assuming that the chemical potential of the individual components in the ideal gas mixture is equal to μ*_i_* = *k*_*B*_ *T* *ln*(*y*_*i*_ *p*), where *y*_*i*_ is the fraction of component *i*,

13 $$\omega_{a}=\omega_{a}\left(y_{1},y_{2},p,v,T\right)=-k_{B}T\int_{0}^{p}\left[N_{1}\left(y_{1},y_{2},p,v,T\right)+N_{2}\left(y_{1},y_{2},p,v,T\right)\right]\frac{1}{p}dp=-\frac{k_{B}T\left(K_{1}N_{1}^{0}y_{1}+K_{2}N_{2}^{0}y_{2}\right)ln\left(1+K_{1}y_{1}p+K_{2}y_{2}p\right)}{K_{1}y_{1}+K_{2}y_{2}}$$

Respectively, the adsorption stress (eq 2) is defined by differentiation of eq 7 with respect to the unit cell volume,

$$\sigma_{a}=-\left(\frac{\partial \omega_{a}}{\partial N_{1}^{0}}\frac{\partial N_{1}^{0}}{\partial v}+\frac{\partial \omega_{a}}{\partial N_{2}^{0}}\frac{\partial N_{2}^{0}}{\partial v}+\frac{\partial \omega_{a}}{\partial K_{1}}\frac{\partial K_{1}}{\partial v}+\frac{\partial \omega_{a}}{\partial K_{2}}\frac{\partial K_{2}}{\partial v}\right),={\mathrm{\sigma ̃}}_{a,1}\frac{K_{1}y_{1}ln\left(1+K_{1}p_{1}+K_{2}p_{2}\right)}{K_{1}y_{1}+K_{2}p_{2}},\lambda N_{1}^{0}+{\mathrm{\sigma ̃}}_{a,2}\frac{K_{2}y_{2}ln\left(1+K_{1}p_{1}+K_{2}p_{2}\right)}{K_{1}y_{1}+K_{2}p_{2}},\lambda N_{2}^{0}-{\mathrm{\sigma ̃}}_{a,1}\left[\frac{y_{1}K_{1}p\left(K_{1}y_{1}+K_{2}y_{2}(N_{2}^{0}/N_{1}^{0}\right))}{\left(1+K_{1}p_{1}+K_{2}p_{2}\right)\left(K_{1}y_{1}+K_{2}y_{2}\right)},+\frac{K_{1}K_{2}y_{1}y_{2}\left(1-(N_{2}^{0}/N_{1}^{0}\right)ln\left(1+K_{1}p_{1}+K_{2}p_{2}\right)}{{\left(K_{1}y_{1}+K_{2}y_{2}\right)}^{2}}\right],\lambda_{K_{1}}-{\mathrm{\sigma ̃}}_{a,2}\left[\frac{y_{2}K_{2}p\left(K_{1}y_{1}\left(N_{1}^{0}/N_{2}^{0}\right)+K_{2}y_{2}\right)}{\left(1+K_{1}p_{1}+K_{2}p_{2}\right)\left(K_{1}y_{1}+K_{2}y_{2}\right)},+\frac{K_{1}K_{2}y_{1}y_{2}\left(1-\left(N_{1}^{0}/N_{2}^{0}\right)\right)ln\left(1+K_{1}p_{1}+K_{2}p_{2}\right)}{{\left(K_{1}y_{1}+K_{2}y_{2}\right)}^{2}}\right]\lambda_{K_{2}}$$ 14

Here, $\frac{\partial N_{i}^{0}}{\partial v}$ and $\frac{\partial K_{i}}{\partial v}$ represent the changes in the maximum adsorption capacity and Langmuir parameter of component *i* = 1,2 with the variation of the unit cell volume *v*. In eq 14, we introduced the dimensional parameters ${\mathrm{\sigma ̃}}_{a,i}$ representing a characteristic magnitude of the adsorption stress exerted by a particular component *i*,

15 $${\mathrm{\sigma ̃}}_{a,i}=\frac{k_{B}TN_{i}^{0}}{v_{0,i}}$$

and the dimensionless parameters, which represent the capacity and Langmuir parameter *susceptibility factors*,

$$\lambda_{N_{i}^{0}}=\frac{\partial N_{i}^{0}}{\partial v}\frac{v_{0,i}}{N_{i}^{0}\left(v_{0,i}\right)}\times 100\%,\mathrm{and},\lambda_{K_{i}}=-\frac{\partial K_{i}}{\partial v}\frac{v_{0,i}}{K_{i}\left(v_{0},i\right)}\times 100\%$$ 16

The susceptibility factors, $\lambda_{N_{i}^{0}}$ and $\lambda_{K_{i}}$ , show the percentage changes of the adsorption capacity and Langmuir parameter induced by the volumetric strain of 1%, taken from the “dry” volume *v*_0,*i*_ of each phase. The susceptibility factors, $\lambda_{N_{i}^{0}}$ and $\lambda_{K_{i}}$ , defined by eq 16 are positive. Using the van’t Hoff equation (eq 12), it is practical to use the adsorption energy susceptibility factor,

16a $$\lambda_{\Delta H_{i}}=\frac{\partial \left(-\Delta H_{i}\right)}{\partial v}\frac{V_{0}}{-\Delta H_{i}\left(V_{0}\right)}=\frac{-\Delta H_{i}}{RT}\lambda_{K_{i}}$$

which can be calculated based on a molecular model of adsorbent structure.

The adsorption stress for pure components can be found from eq 14 by setting the respective mole fraction *y*_i_ = 1

17 $$\sigma_{a,i}={\mathrm{\sigma ̃}}_{a,i}\left[-\frac{K_{i}p}{1+K_{i}p}\lambda_{K_{i}}+ln\left(1+K_{i}p\right)\lambda_{N_{i}^{0}}\right]$$

Noteworthy, eq 18 for single component adsorption, albeit in a different form, was derived and employed earlier for modeling breathing transitions in MIL-53 MOFs.^19^

Equations 14 and 18 allow for a qualitative analysis of the variation of the adsorption stress in the process of adsorption. The adsorption stress is determined by a competition of two factors upon the increase of the pore volume: (1) the decrease of the adsorption energy and, respectively, the decrease of the Langmuir parameter, *k*_*i*_, and (2) the increase of the adsorption capacity, $N_{i}^{0}$ . At low pressures, *k*_*i*_ *p* ≪ 1, where the adsorption stress, $\sigma_{a,i}\approx -{\mathrm{\sigma ̃}}_{a,i}K_{i}p\left[\lambda_{K_{i}}-\lambda_{N_{i}^{0}}\right]$ . If $\lambda_{K_{i}}>\lambda_{N_{i}^{0}}$ , the first factor dominates, and the adsorption stress is negative and causes adsorbent contraction. This is the most common case for microporous adsorbents.^13,20,36^ At high pressures, *k*_*i*_ *p* ≫ 1, the second factor dominates, and the adsorption stress is positive and increases logarithmically, $\sigma_{a,i}\approx {\mathrm{\sigma ̃}}_{a,i}ln\left(K_{i}p\right)\lambda_{N_{i}^{0}}$ , causing adsorbent expansion/swelling. The adsorption stress minimum, which corresponds to the maximum contraction, is achieved at $p_{m,i}={K_{i}}^{-1}\left[\lambda_{K_{i}}-\lambda_{N_{i}^{0}}\right]/\lambda_{N_{i}^{0}}$ . The respective minimum stress and maximum contraction strain equal, respectively, $\sigma_{m,i}=-{\mathrm{\sigma ̃}}_{a,i}\left[\lambda_{K_{i}}-(1+ln\left(\lambda_{K_{i}}/\lambda_{N_{i}^{0}}\right)\lambda_{N_{i}^{0}}\right]$ and $\varepsilon_{m,i}=-\frac{1}{k}\{{\mathrm{\sigma ̃}}_{a,i}\left[\lambda_{K_{i}}-(1+ln\left(\lambda_{K_{i}}/\lambda_{N_{i}^{0}}\right)\lambda_{N_{i}^{0}}\right]-{K_{i}}^{-1}\left[\lambda_{K_{i}}-\lambda_{N_{i}^{0}}\right]/\lambda_{N_{i}^{0}}\}.$

The general equations of the Langmuir model (eqs 11 and 14) allow for prediction of the adsorption and stress isotherms in the process of binary adsorption at different mixture compositions, pressures, and temperatures based on the parameters fitted to the experimental data on pure component adsorption and strain isotherms measured at one temperature.

#### Equation for the Adsorption Stress in Process of Adsorbate Exchange

Experiments on CH_4_ displacement by CO_2_ ^12^ were performed by changing the CO_2_ fraction, *y*_1_, in the mixture at a fixed pressure *p* starting from pure CH_4_, *y*_1_ = 0 and *y*_1_ = 1, to pure CO_2_, *y*_1_ = 1 and *y*_2_ = 0. The respective change of the adsorption potential, Δ*ω*_*a*_ can be calculated as the difference between the adsorption potentials of the CH_4_–CO_2_ mixture at given *y*_1_ (*y*_2_ = 1-*y*_1_) and *p* and pure CH_4_ at given pressure *p*,

18 $$\Delta \omega_{a}=\omega_{a}\left(y_{1},y_{2},p,v,T\right)-\omega_{a}\left(0,1,p,v,T\right)=-\frac{k_{B}T\left(K_{1}N_{1}^{0}y_{1}+K_{2}N_{2}^{0}y_{2}\right)\ln\left(1+K_{1}y_{1}p+K_{2}y_{2}p\right)}{K_{1}y_{1}+K_{2}y_{2}}+k_{B}TN_{2}^{0}\ln\left(1+K_{2}p\right)$$

Respectively, the adsorption stress, Δσ_a_(*y*_1_,*y*_2_,*p,v,T*), induced by CH_4_ displacement at fixed pressure *p* is defined by differentiation of eq 19 with respect to *v*,

19 $${\Delta \sigma }_{a}\left(y_{1},y_{2},p,V,T\right)=\sigma_{a}\left(y_{1},y_{2},p,v,T\right)-\sigma_{a,2}\left(p,v,T\right)$$

Note that σ_a,2_(*p,v,T*) is the adsorption stress induced by pure CH_4_ at pressure *p*. Using the volumetric modulus *k*, the stress isotherm can be converted into the strain isotherm, counted from the state of pure CH_4_ at pressure *p*,

20 $$\Delta \varepsilon ={\Delta \sigma }_{a}/k$$

Here, σ_a_ and σ_a,2_ are determined by eqs 10 and 11 and represent, respectively, the adsorption stress upon CH_4_–CO_2_ mixture and pure CH_4_ adsorption at pressure *p,* both counted from the “dry” LP phase at *p* = 0. Equations 20 and 21 represent the stress and strain isobar-isotherms as functions of the mixture composition at constant pressure *p*. The strain isotherm is a physical observable, and can be compared with the experimental one if the latter is available.^34,37^

#### Model Parametrization

The model proposed above depends on two sets of four parameters characterizing adsorption properties of the individual components in both the NP and LP phases: the adsorption capacities and Henry constants, $N_{i}^{0}$ and *K*_*i*_, and the adsorption capacity and Langmuir parameter susceptibility factors, $\lambda_{N_{i}^{0}}$ and $\lambda_{K_{i}}$ . The parametrization of the Langmuir model in both phases is performed by relying on single adsorption–desorption isotherm experiments, where different pressure ranges are associated with the NP and LP phases. Provided that the experimental single component adsorption and strain isotherms are available, the model parametrization is straightforward. First, the experimental adsorption isotherms of pure components are approximated by the one component Langmuir equations,

21 $$N_{i}=\frac{N_{i}^{0}K_{i}p}{1+K_{i}p}$$

to determine the adsorption capacities, $N_{i}^{0}$ , and Henry constants, *K*_i_ Next, the susceptibility factors, $\lambda_{N_{i}^{0}}$ and $\lambda_{K_{i}}$ , are determined by fitting the experimental pure component strain isotherms using eq 8 with the adsorption stress σ_*a,i*_ given by eq 18. The volumetric modulus, *k*, in eq 8 has to be determined from independent compression experiments, e.g., by mercury porosimetry.^13,38^ However, this ideal parametrization strategy, which requires experimental data on both adsorption and strain isotherms of the individual components, has several caveats.

First, it should be noted that the adsorption potential, ω_*a*_, defined by eq 13 and, respectively, the stress in eqs 14 and 18 are counted from the idealized “dry” sample that is fully evacuated at *p* = 0. In experiments, such a completely “dry” state is never achieved due to inevitable residuals. The experimental “dry” state, which corresponds to the lowest pressure recorded in the beginning adsorption measurement (before the first point of the adsorption isotherm), depends on the conditions of sample preparation and is prestressed compared to the idealized “dry” state. This prestress must be taken into account while comparing the experimental strain isotherm with the theoretical prediction according to eq 8. Since the ideal “dry” state cannot be accessed, it is practical to count the strain from an accessible state at nonzero pressure, p*, that can be chosen as the first point of the strain isotherm that is reliably measured, i.e., at *p* = p*. To this end, either the experimental or the theoretical strain isotherm should be shifted to ensure that both isotherms coincide at *p* = p* Alternatively, the state of complete saturation for vapors, or at the highest measured pressure for supercritical fluids, can be chosen as the reference state from which the adsorption potential and stress are reckoned. This approach was used in predicting adsorption deformation of activated carbon upon benzene and *n*-hexane adsorption.^33^

In the case of breathing transitions in MOFs, the parametrization is complicated since the experimental adsorption isotherms in NP and LP phases are measured within limited ranges of pressures, which correspond to the regions of phase metastability. Also, the choice of the reference state for the NP phase is ambiguous because, at the “dry” conditions, only the LP phase of MIL-53 is stable: therefore, adsorption data in the NP phase at low pressures cannot be accessed experimentally.

The main problem is that consistent experimental data on strain isotherms is rarely available. Due to these constraints, parametrization of the susceptibility factors can instead be based on certain assumptions about the adsorption and deformation processes. This is the case with the studies of breathing transitions in flexible MOFs, where the steps on the adsorption isotherms are assumed to correspond to the NP-LP phase transitions and the positions of these steps are used for fitting the theoretical stress isotherms.^19^ The position of the NP-LP phase equilibrium is not measurable and is located somewhere in the middle of the hysteresis loop formed by adsorption–desorption isotherms. It can be determined theoretically assuming the equality of the osmotic potentials in the coexisting NP and LP phases.^14^

According to the threshold stress ansatz,^19^ one assumes that the onset of the transition occurs upon achieving the maximum (threshold) stress, σ_t_, and, respectively, the maximum strain, ε_t_, that the given phase can resist before deforming elastically. It was shown that this threshold stress can be directly measured in the mercury porosimetry compression–expansion experiments.^13,38^ As applied to multicomponent adsorption, we further assume that the threshold stress is a property of the host matrix and is independent of the nature of the adsorbate. This assumption allows us to define the threshold stresses and strains for NP-LP and LP-NP transitions from the adsorption experiments with the pure adsorbate (i.e., CO_2_), for which the respective experimental data on the adsorption–desorption cycles is available.

## Results

### Breathing Transitions upon Displacement of CH_4_ by CO_2_ on MIL-53­(Al)

The proposed theoretical approach is tested on the experimental data of Ortiz et al.^12^ on deformation of MIL-53­(Al) caused by the displacement of CH_4_ and CO_2_. The goal is to validate the hypothesis that by using the parameters determined from the fitting of pure component adsorption experiments, one can predict the experimental observations in the course of mixture adsorption. We focus specifically on the temperatures 273 K and higher, at which adsorption of pure CH_4_ proceeds in the LP phase without transitions to the NP state, while addition of CO_2_ in the adsorbate mixture triggers the LP-NP transition at low pressures and consequent NP-LP transition at higher pressure, qualitatively similar to that displayed for pure CO_2_.

### Model Parametrization Based on Pure Component Experimental Data

The adsorption deformation model for binary mixtures, eqs 11 and 14, requires a total of 16 parameters for the adsorption capacity, $N_{{\mathrm{CO}}_{2}}^{0}$ and $N_{{\mathrm{CH}}_{4}}^{0}$ , Henry constant, $K_{{\mathrm{CO}}_{2}}$ and $K_{{\mathrm{CH}}_{4}}$ and the susceptibility factors, $\lambda_{N_{{\mathrm{CO}}_{2}}^{0}}$ , $\lambda_{K_{{\mathrm{CO}}_{2}}},\lambda_{N_{{\mathrm{CH}}_{4}}^{0}}$ , and $\lambda_{K_{{\mathrm{CH}}_{4}}}$ in both NP and LP phases,. These parameters determined from the individual adsorption data are presented in Table 1.

**Table 1:** Langmuir Adsorption and Stress Model Parameters at 273 K

Species/phase	*N*^0^(molec/u.c)	*K* (bar^–1^)	σ̃_*a,i*_	*p_m,i_* (bar)	σ*_m,i_* (bar)	λ_N^0^_	λ*_K_*
CO_2_ / NP	2.7	18.4	97	0.24	–261	1	–5.37
CO_2_ / LP	9.81	0.68	249	0.73	–57	2.45	–3.66
CH_4_ / NP	3.92	0.46	141	9.5	–379	1	–5.37
CH_4_/ LP	6.49	0.18	164	2.73	–38	2.45	–3.66

The adsorption capacity and Langmuir parameters for CH_4_ adsorption in the LP phase, $N_{{\mathrm{CH}}_{4},LP}^{0}$ and $K_{{\mathrm{CH}}_{4},\mathrm{LP}}$ , were taken from ref. 18 The adsorption capacity and Langmuir parameters for CO_2_ adsorption in the NP and LP phases, $N_{{\mathrm{CO}}_{2},NP/LP}^{0}$ and $K_{CO_{2},NP/LP}$ , were obtained by direct fitting of the experimental data from ref. 12 at 273 K, since they vary slightly from those included in ref. 18 (see Figure S2 of ref. 12). Since the experimental data at 273 K is not available, the parameters for CH_4_ adsorption in the NP phase, $N_{{\mathrm{CH}}_{4},NP}^{0}$ and $K_{{\mathrm{CH}}_{4},NP}$ , were obtained by extrapolating the Langmuir parameters from ref. 18 to 273 K. The respective pure component Langmuir isotherms are presented in Figure 2 (1st panel) for CO_2_ and in Figure S1 for CH_4_ in comparison with the experimental data.

**Figure 2:**
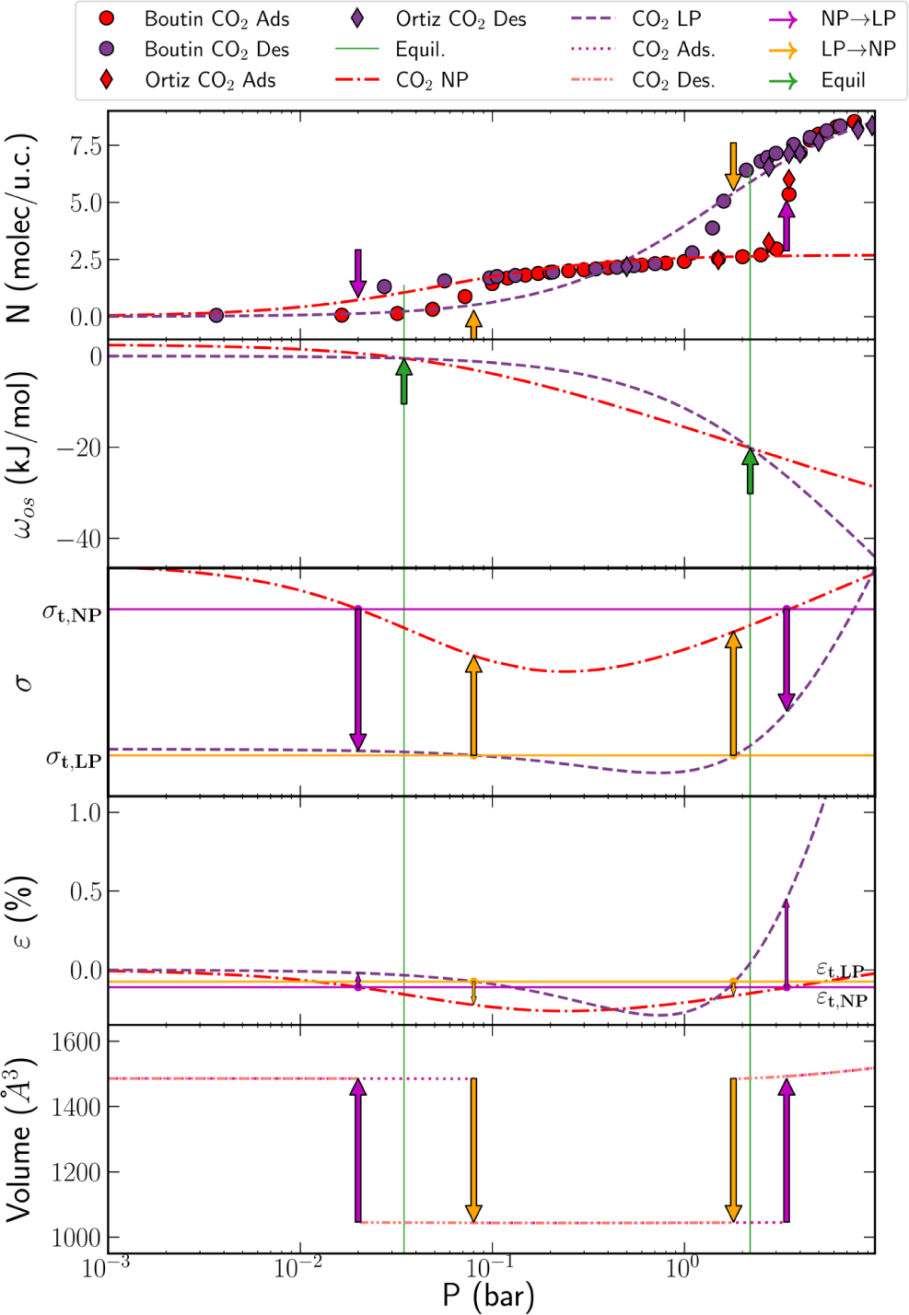
1st row: Experimental adsorption–desorption isotherms of pure CO_2_ on MIL-53­(Al) at 273 K and the Langmuir approximation of the isotherms in NP and LP phases.^18^ Green lines and arrows show the positions of low-pressure and high-pressure NP-LP phase equilibrium, *p*_*e,l*_ and *p*_*e,h*_, purple and yellow arrows correspond to the threshold pressures of the NP-LP and LP-NP transitions, *p*_NP,*l/h*_ and *p*_LP,*l/h*_, respectively. 2nd row: Calculated osmotic potentials of the NP and LP phases, $\omega_{os}^{NP}$ and $\omega_{os}^{LP}$ ; $\omega_{os}^{NP}$ is shifted up by the difference in the framework free energies of empty NP and LP phases, $\Delta f_{s,0}=f_{s,0}^{NP}-f_{s,0}^{LP}$ . The intersections correspond to the positions of NP-LP phase equilibrium located inside the hysteresis loop. 3rd row: Predicted stress isotherms. Horizontal purple (σ_*t*_,*_NP_* NP-LP) and yellow (σ_*t*_,*_LP_* LP-NP) lines correspond to the equality of the threshold stresses. 4th row: Predicted volumetric strain isotherms calculated using the volumetric moduli of NP and LP phases, *k* ^*NP*^ = 10 GPa and *k* ^*LP*^ = 2 GPa. Horizontal lines correspond to the equal threshold strains at the low-pressure and high-pressure NP-LP (ε_t,NP_, purple) and LP-NP (ε_t,LP_, yellow) transitions. 5th row: Change in the unit cell volume along the adsorption and desorption isotherms.

Since the strain isotherms in the system considered are not available, the parametrization of the susceptibility factors was performed based on the analysis of the CO_2_ adsorption–desorption isotherm at 273 K (Figure 2, first row). This isotherm consists of two hysteresis loops corresponding to low-pressure and high-pressure NP-LP and LP-NP transitions. First, we invoke the condition of equality of the osmotic potentials and require that the pressures, *p*_*e,l*_ and *p*_*e,h*_, of the low-pressure and high-pressure NP-LP phase equilibrium predicted by eq 10 must be located in the middle of the hysteresis loop. The respective equilibrium pressures are estimated as *p*_*e,l*_ = 0.05 bar and *p*_*e,h*_ = 2.6 bar. This requirement yields 2 conditions; however, it involves an additional unknown, $\Delta f_{s,0}=f_{s,0}^{NP}-f_{s,0}^{LP}$ , the difference in the framework free energies of empty NP and LP phases. The equilibrium conditions correspond to the intersection of the calculated osmotic potentials of the NP and LP phases, $\omega_{os}^{NP}$ and $\omega_{os}^{LP}$ , presented in in Figure 2 (2nd panel). Here, the osmotic potential of the LP phase, $\omega_{os}^{LP}$ , is measured from the empty LP phase, $f_{s,0}^{LP}$ and the osmotic potential of the NP phase, $\omega_{os}^{NP}$ , is shifted up by Δ*f*_*s,*0._ The value of Δ*f*_*s*,0_ = 2.5 kJ/mol corresponds to the one estimated by Coudert et al.^22^ It is worth noting that eq 10 for the osmotic potential includes the elastic contribution that is determined by the adsorption stress calculated by eq 18, which depends on the susceptibility factors, $\lambda_{N_{{\mathrm{CO}}_{2}}^{0}}$ and $\lambda_{K_{{\mathrm{CO}}_{2}}}$ . However, this contribution is of the second order with respect to the strain and is insignificant within the elastic regime of deformation with the strains not exceeding several percents.^13^

Second, we assume that the experimentally observed phase transitions correspond to the steps on of the adsorption–desorption hysteresis loops and invoke the threshold stress hypothesis that requires the equality of the threshold stresses and strains of the low-pressure and high-pressure NP-LP and LP-NP transitions,

22a $$\sigma_{\mathrm{NP}}\left(p_{\mathrm{NP},l}\right)=\sigma_{\mathrm{NP}}\left(p_{\mathrm{NP},h}\right)=\sigma_{t,\mathrm{NP}},\mathrm{and},\sigma_{\mathrm{LP}}\left(p_{\mathrm{LP},l}\right)=\sigma_{\mathrm{LP}}\left(p_{\mathrm{LP},h}\right)=\sigma_{t,\mathrm{LP}}$$

22b $$\varepsilon_{\mathrm{NP}}\left(p_{\mathrm{NP},l}\right)=\varepsilon_{\mathrm{NP}}\left(p_{\mathrm{NP},h}\right)=\varepsilon_{t,\mathrm{NP}},\mathrm{and},\varepsilon_{\mathrm{LP}}\left(p_{\mathrm{LP},l}\right)=\varepsilon_{\mathrm{LP}}\left(p_{\mathrm{LP},h}\right)=\varepsilon_{t,\mathrm{LP}}$$

Here, σ_t,NP_ and σ_t,LP_ and ε_t,NP_ and ε_t,LP_ are, respectively, the threshold stresses and strains that can be withheld by NP and LP phases before the onset of the spontaneous plastic deformation and the transition into LP and NP phases, respectively. The equalities (eqs 23,24) are shown by the horizontal lines in Figure 2 (3rd panel), which presents the stress isotherms calculated by eqs 6 and 18. The threshold stresses correspond to the inflection points of the adsorption and desorption steps of the respective hysteresis loops marked by purple and yellow arrows. The respective pressures were estimated as *p*_NP,*l*_= 0.02 bar,*p*_NP,*h*_= 3.4 bar, *p*_LP,*l*_ = 0.08 bar, and *p*_LP,h_ = 1.8 bar It is worth noting that, since the empty NP phase does not exist at the experimental conditions, the magnitude of this prestress represents the additional unknown, which is chosen here as 450 bar, and the calculated stress in the NP phase is shifted by this value· Noteworthy, at the conditions of adsorption experiments considered here, the adsorption stress significantly exceeds the gas pressure, so that the contribution of the gas pressure into the stress determined by eq 6 can be neglected. The threshold stresses are estimated as σ_t,NP_ = 340 bar and σ_t,LP_ = −15 bar A smaller absolute value for the threshold stress for the LP-NP transition is explained by a 5-fold difference in the volumetric modulus–the NP phase is much “stiffer” than the LP phase. The respective threshold strains are ε_t,NP_ = 0.34% and ε_t,LP_ = 0.075%.

The third panel in Figure 2 presents the predicted stress isotherms, and the fourth panel represents the corresponding strain isotherms calculated using the volumetric moduli of NP and LP phases, *k* ^*NP*^ = 10 GPa and *k* ^*LP*^ = 2 GPa.^13^ The hysteretic phase transitions are approximated by vertical steps at the determined threshold pressures. The fifth panel of Figure 2 presents the variation of the unit cell volume in the process of adsorption and desorption. Starting from the reference volume $v_{LP}^{0}$ of the empty LP phase, it decreases as $v\left(p\right)=v_{LP}^{0}\left(1+\frac{1}{k^{LP}}\sigma_{\mathrm{LP}}\left(p\right)\right)$ and the framework contracts. Upon achieving the threshold stress σ_t,LP_ at *p* = *p*LP-NP,_*l*_, the LP-NP transition occurs with a stepwise decrease of the unit cell volume to $v_{NP}^{0}(1+\frac{1}{k^{NP}}\sigma_{\mathrm{NP}}\left(p_{\mathrm{LP}-\mathrm{NP},l}\right))$ . Then the framework deforms elastically in the NP phase, as $v\left(p\right)=v_{NP}^{0}(1+\frac{1}{k^{NP}}\sigma_{\mathrm{NP}}\left(p\right))$ , until the threshold stress σ_t,NP_ is achieved at *p* = *p*_NP‑LP,h_ and the NP-LP transition occurs with a stepwise increase of the unit cell volume to $v_{LP}^{0}(1+\frac{1}{k^{LP}}\sigma_{\mathrm{LP}}\left(p_{\mathrm{NP}-\mathrm{LP},h}\right))$ . As pressure increases further, the LP phase expands elastically. Along the desorption branch, the consecutive stepwise LP-NP and NP-LP transitions take place at *p* = *p*_LP,h_ and *p* = *p*_NP,l_, respectively.

The susceptibility factors were parametrized by first assuming that $\lambda_{N^{0},NP}=1$ and $\lambda_{N^{0},LP}=2.45$ , which denotes a 1% and 2.45% change, respectively, in the adsorption capacity with 1% change in volume. The Langmuir parameter susceptibility factors, λ*_K_*, for CO_2_ were then obtained by considering the positions of the phase transitions in the experimental adsorption isotherms: the threshold stress σ_*t*_,*_NP_*_/_ *_LP_* necessary for the structure to undergo a phase transition upon adsorption is equal to the stress necessary for the structure to experience the reverse transition upon desorption. By invoking this threshold stress, the values of λ*_K_* necessary to achieve the threshold stress at the positions of the transition were determined. Parameterization of the adsorption stress model for pure CH_4_ is complicated by the fact that CH_4_ adsorption at 273 K and higher temperatures proceeds only in the LP phase. Similar to the approach used in ref. 18, the susceptibility factors for pure CH_4_ were taken equal to those of CO_2_, $\lambda_{N_{CH_{4},i}^{0}}=\lambda_{N_{CO_{2},i}^{0}}$ and $\lambda_{K_{CH_{4},i}}=\lambda_{K_{CO_{2},i}}$ .

The parameters determined from the pure adsorption experimental data are presented in Table 1. Table 1 also presents the predictions for the point of maximum contraction. Note here that σ*_m,i_* represents the maximum magnitude of the adsorption stress for each species and phase counted from the state of zero stress in the LP phase, that is, the NP phase is not shifted by the prestress mentioned above. This is done to highlight the relative magnitude of the contraction in each phase. For pure CO_2_ adsorption, the magnitude of the adsorption stress at the point of maximum contraction is significantly higher in the NP phase than in the LP phase. This is due to the tighter packing of fluid in the collapsed structure. Note also that the pressure at which the maximum contraction occurs in the NP phase is also lower than in the LP phase. The relative trends for the CH_4_ NP and LP phases are similar to the CO_2_ LP and NP phases. The magnitude of the adsorption stress at maximum contraction for CH_4_ in the LP phase is lower than that of CO_2_ in the LP phase, and the pressure at which this maximum contraction is achieved is significantly higher. This is due to the increased adsorption affinity for CO_2_ on MIL-53­(Al) than for CH_4_.

### Prediction of Adsorption Deformation upon CH_4_ Displacement by CO_2_

Using the parametrization of the single component CH_4_ and CO_2_ adsorption and stress isotherms (Table 1), we present in Figure 3 the results of the proposed model of binary adsorption. The first panel presents experimental adsorption isobar-isotherms of CH_4_ displacement by CO_2_ at 273 K as a function of the CO_2_ fraction in the gas phase at constant gas pressure, *p* = 2.75, 3.5, 4, and 5 bar. The lines show the respective isotherms in NP and LP phases predicted by the binary Langmuir model, eq 11. All isobar-isotherms presented start at $y_{{\mathrm{CO}}_{2}}=0$ from the pure CH_4_ adsorption in the LP phase at given pressure. Addition of CO_2_ causes the transition to the NP phase. Then, as the CO_2_ fraction increases the adsorption proceeds in the NP phase until the NP-LP transition occurs. As *p* = 3.5 bar is about the NP-LP transition pressure estimated for the pure CO_2_ adsorption (Figure 2), this transition take place at the CO_2_ fraction close to 1. For higher pressures (4 and 5 bar) the transition occurs at lower CO_2_ fractions. This observation suggests the NP-LP transition mainly depends on the amount of adsorbed CO_2_ rather than on the gas pressure—in line with the stronger interaction of CO_2_ with the host matrix than that of CH_4_. Upon the decrease of the CO_2_ fraction in the LP phase, the isobar follows the prediction of the binary Langmuir model forming a prominent hysteresis loop. The transition to the NP phase occurs at a significantly lower CO_2_ fraction than that of the NP-LP transition. As the gas pressure decreases the hysteresis loop widens.

**Figure 3:**
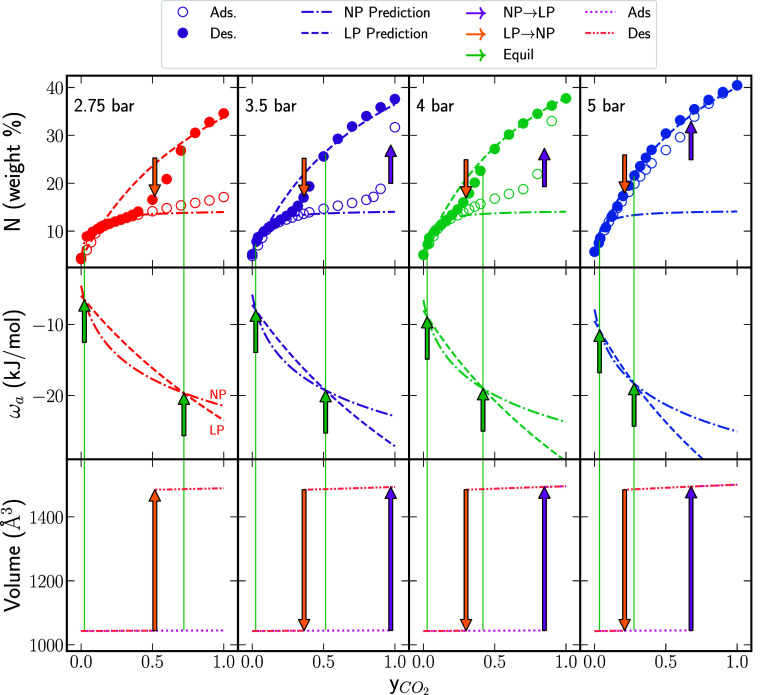
1st row: Experimental adsorption isobar–isotherms^12^ for several pressures at 273 K during CO_2_ displacement of CH_4_ plotted with the Langmuir model predictions for the NP and LP phases. 2nd row: Calculated osmotic potentials in the NP and LP phases. 3rd row: Changes in volume during CO_2_ displacement of CH_4_ resulting from the volumetric strain.

The position of NP-LP and LP-NP transitions are determined following the threshold stress hypothesis.^19^ We assume that disregarding of the gas mixture composition, the phase transition induced by CO_2_ displacement of CH_4_ occurs upon achieving the threshold stress, σ_t,NP_ or σ_t,LP_, characteristic for the phase transformation induced by pure CO_2_. This assumption stems from the hypothesis that the threshold stress is inherent to the host matrix and does not depend on the nature of the guest phase. As such, we predict for any given gas mixture, provided the pressure being kept constant, the fraction of CO_2_, at which the stress in the NP phase, determined by eqs 8 and 14, equals the threshold stress, σ_t,NP_ = 340 bar, of the NP-LP phase transition in the case of pure CO_2_ adsorption. The position of the LP-NP phase transition during depletion of the CO_2_ concentration in the gas mixture is determined similarly.

The position of NP-LP phase equilibrium located inside the hysteresis loop is determined by the equality of the osmotic potentials of the NP and LP phases, $\omega_{os}^{NP}\left(y_{{\mathrm{CO}}_{2},e},p\right)=\omega_{os}^{LP}\left(y_{{\mathrm{CO}}_{2},e},p\right)$ , eq 20. The calculated osmotic potentials are shown in the second panel of Figure 3. The third panel presents the variation of the unit cell volume as a function of the CO_2_ fraction.

The calculated CO_2_ fractions corresponding to the NP-LP and LP-NP phase transitions are reported in Table 2, and the corresponding adsorption and stress isobar-isotherms at different gas mixture pressures at 273 K are plotted in Figure 3.

**Table 2:** Stresses and Corresponding CO_2_ Fractions Necessary to Induce Phase Transitions along the Mixture Isobars at 273 K

P (bar)	1.8	3.4	3.5	4	5
σ*_LP‑NP_* (bar)	–15	–15	–15	–15	–15
$y_{{\mathrm{CO}}_{2},LP‐NP}$	1	0.38	0.36	0.3	0.21
σ*_NP‑LP_* (bar)	-	340	340	340	340
*y*_CO_2,_ *_NP‑LP_*_	-	1	0.97	0.85	0.68

There are a few features in Figure 3 worth noting. First, the isobar-isotherm of the CO_2_/CH_4_ mixture at 273 K and 2.75 bar does not exhibit the NP-LP transition during displacement of CH_4_ by CO_2_. The stress model accounts for this lack of transition, as the system at these conditions never achieves the NP-LP threshold stress of 340 bar—staying in a metastable NP state. For all other pressures, the NP-LP transition (yellow arrows) and LP-NP transition (purple arrows) are visualized. As the gas pressure increases, the fraction of CO_2_ necessary to achieve both the NP-LP threshold stress and the LP-NP threshold stress decreases.

The proposed model parametrized based on the experimental data at a certain temperature (here at 273 K) can be utilized to predict the adsorption deformation at other temperatures by rescaling the model parameters using the van’t Hoff equation (eq 12). The latter implies that the susceptibility factors *λ*_*K,i*_ (eq 16) at different temperatures fulfill the following relationship, $\lambda_{K,i}\left(T_{1}\right)=\frac{T_{2}}{T_{1}}\lambda_{K,i}\left(T_{2}\right)$ . An example of such calculations for the case of CO_2_/CH_4_ mixture at 253 K is given in Supporting Information.

## Conclusions

We present a general thermodynamic approach to modeling the effects of adsorption-induced deformation of nanoporous materials in case of multicomponent gas mixtures. The approach is based on the concept of the adsorption stress exerted by the guest molecules of the host porous matrix. This adsorption stress approach was found instrumental for the description of single component adsorption systems, particularly for the analyses of the breathing transitions between narrow pore NP and large pore LP phases of MIL-53 MOF.^19^ This system demonstrates two consecutive transformations in the course of adsorption. At low gas pressures, the adsorption proceeds in the LP phase. Upon the increase of pressure, the LP phase becomes unstable and transforms into the NP phase, which becomes unstable and transforms back to the NP phase in high pressures. On the desorption pass, a significant hysteresis is with two reverse phase transformations is observed. The onset of PL-NP and NP-LP phase transformations is characterized by the respective threshold adsorption pressures, which the structure cannot resists. It was assumed that the threshold tress magnitudes are the characteristic of the framework and do not depend on the nature of the stimulus and the type of the gas.

Here, we extend the adsorption stress approach for modeling binary adsorption of CH_4_ and CO_2_ and study the breathing transition triggered by the displacement of adsorbed CH_4_ by CO_2_, which was explored in earlier experiments by Ortiz et al.^12^ on deformation of MIL-53­(Al) at different temperatures. This system exhibits an interesting behavior that has not been theoretically explained. While the adsorption of pure CH_4_ at the temperatures 273 K and higher proceeds in the LP phase without transitions to the NP state, the addition of CO_2_ in the adsorbate mixture triggers the LP-NP transition at low pressures and consequent NP-LP transition at higher pressure, qualitatively similar to that displayed for pure CO_2_. The phase transition positions depend on the CO_2_ fraction, total gas mixture, and temperature.

Using the Langmuir model of binary adsorption, we derive the equations for the adsorption stress with the parameters defined for the single adsorption and strain isotherms. Within this model, the adsorption stress accounts for the impact of the variation of the framework volume to due to an applied stimulus on the energy of adsorption and adsorption capacity of flexible structures by introducing the respective susceptibility factors. We find that the proposed model qualitatively describes the phase transformations, which occurs by increasing the CO_2_ fraction at the constant pressure and temperature of the CH_4_–CO_2_ mixture. A proper parametrization based on the single component measurements allowed for a semiquantitative agreement between the theoretically predicted and experimental adsorption and strain isotherms within the measured ranges of gas pressure and temperature.

As the *in situ* experimental studies of adsorption-induced deformation are complicated and require an expensive instrumentation, the theoretical models can inform the design of flexible nanoporous structures for particular applications that involve gas mixtures. The proposed general approach for accounting for adsorbent deformation during adsorption of gas mixtures is applicable to diverse systems of practical interest such as adsorption separations, gas and energy storage, CO_2_ capture, atmospheric water harvesting, and secondary gas recovery and carbon dioxide sequestration in coal and shale reservoirs.

## Supplementary Material



## Notes

Experimental adsorption–desorption isotherms of CH_4_; experimental and predicted adsorption–desorption and strain isotherms for pure CO_2_ at 254 K and CH_4_ at 250 K; experimental adsorption and strain isobar–isotherms at two pressures for a mixture of CH_4_ and CO_2_ at 253 K; the adsorption isobar–isotherms for *p* = 3.5 bar (PDF)
