# Supplementary material for: Phase Transformations in MOFs Induced by Adsorbate Exchange
Source: Langmuir. 2025 Feb 17;41(7):4720–9. doi: 10.1021/acs.langmuir.4c04626 (PMC12333355; doi:10.1021/acs.langmuir.4c04626)
Supplement: Supplementary file 1 [file la4c04626_si_001.pdf]

**Phase Transformations in MOFs Induced by Adsorbate Exchange**

Alexander V. Neimark[1,*], Nicholas J. Corrente[1], and François-Xavier Coudert[2]

1) Department of Chemical and Biochemical Engineering, Rutgers, The State University of New Jersey, 98 Brett Road, Piscataway, NJ 08854, USA.

2) Chimie Paris Tech, PSL University, CNRS, Institut de Recherche de ChimieParis, 11 Pierre and MarieCurie, 75231 Paris, France.

## Supporting Information

Number of pages: 3

Number of figures: 4

Figure S1: Experimental adsorption-desorption isotherms of CH4 on MIL-53 (AL) at 273K and the Langmuir approximation of the isotherms in NP and LP phases. ..........................................S1

Figure S2: Experimental[1] and predicted adsorption-desorption (top) and strain (bottom) isotherms for pure $CO_2$ at 254 K and $CH_4$ at 250 K. ....................................................................S2

Figure 3: Experimental adsorption[2] (top) and strain (bottom) isobar-isotherms at two pressures for a mixture of CH4 and CO2 at 253 K. ..........................................................................S2

Figure S4: The adsorption isobar-isotherms for p=3.5 bar from in Figure 3 of the main text presented in semi-logarithmic scale to show clearly the phase transitions ..................................S3

* Corresponding author: aneimark@rutgers.edu

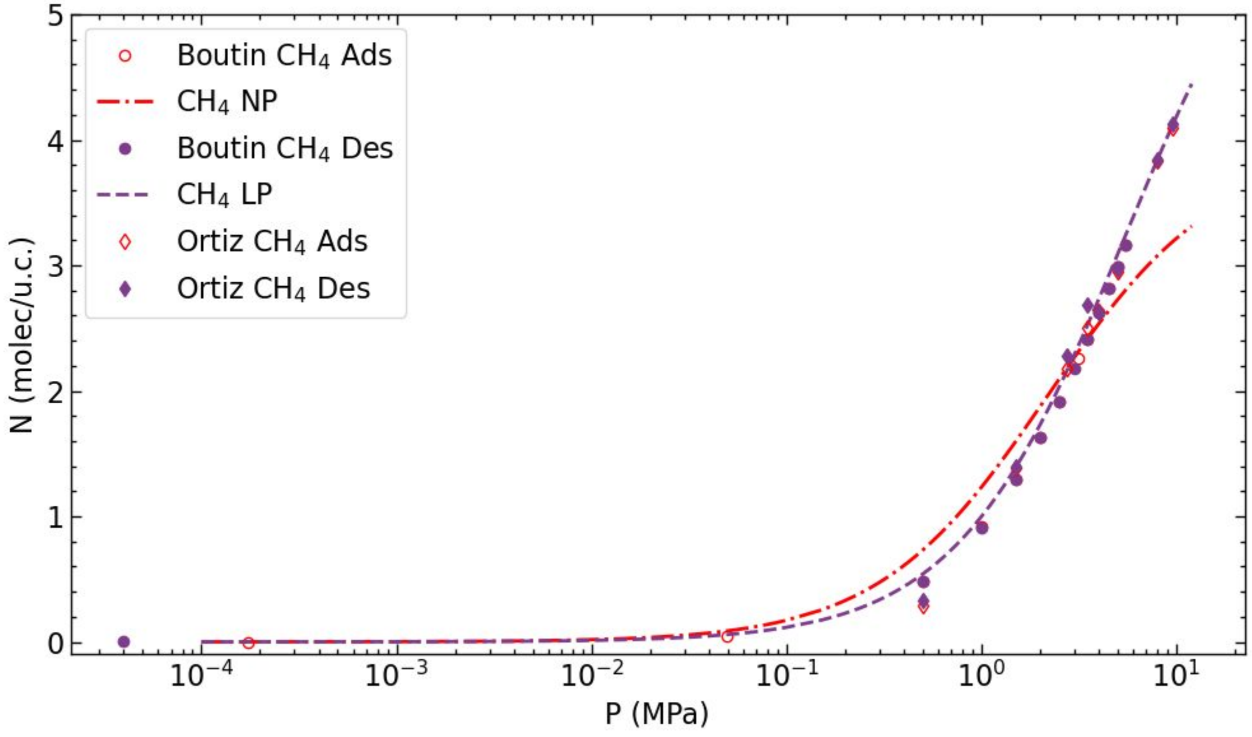


*Figure S1: Experimental adsorption-desorption isotherms of CH4 on MIL-53 (AL) at 273K and the Langmuir approximation of the isotherms in NP and LP phases.*

We have performed this same procedure for a CO2/CH4 mixture at 253 K. We parameterize the Langmuir adsorption model by fitting the experimental data for $CO_2$ at 254 K and $CH_4$ at 250 K from Ref. [1]. We use the same adsorption capacity susceptibility factors $\lambda_{N^0,i}$ for each phase at 273 K, because we assume that they do not vary significantly with temperature. We rescale the Langmuir parameter susceptibility factor $\lambda_{K,i}$ for each phase by employing the van't Hoff equation, i.e. $\lambda_{K,i}(T_1) = \frac{T_2}{T_1}\lambda_{K,i}(T_2)$. The results of this analysis are shown in Figures S2 and S3 and Tables S2 and S3. Although the hysteresis regions are less defined at 253 K than at 273 K, the model still shows reasonable predictions for the positions of the phase transitions.

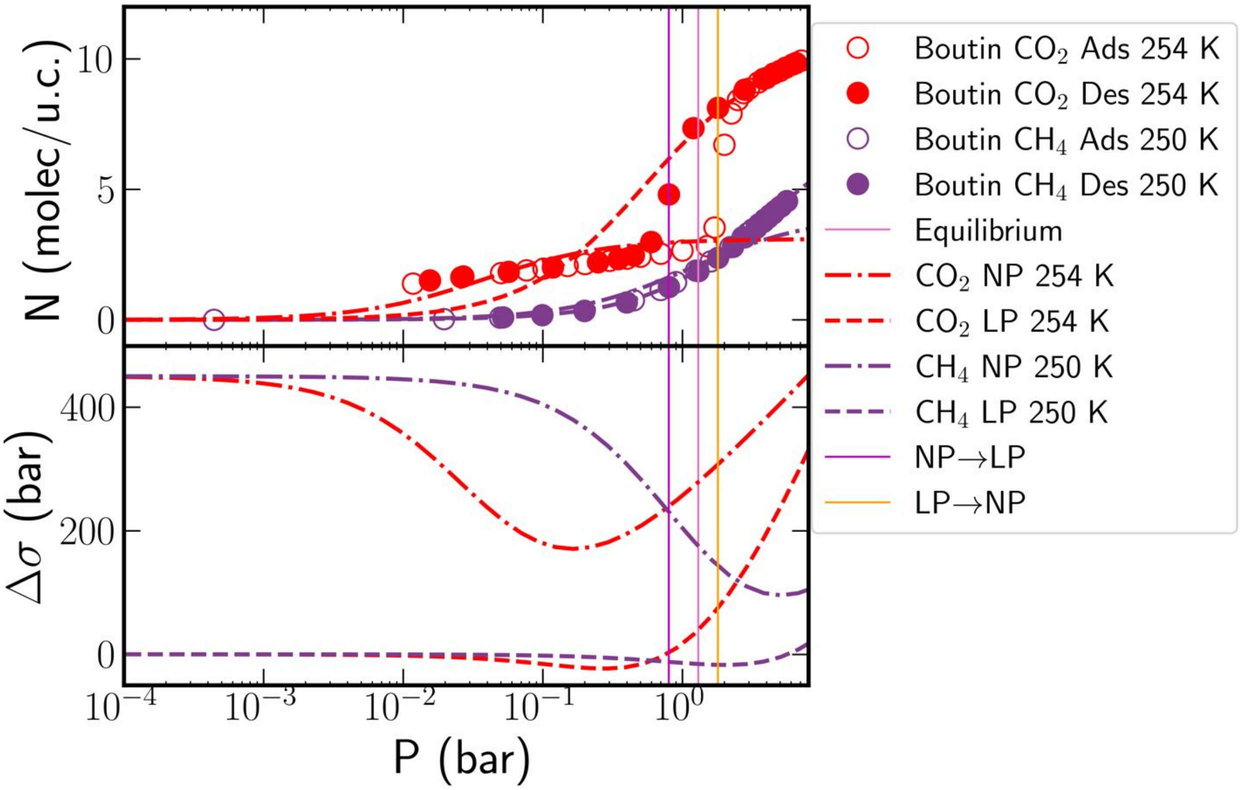


*Figure S2: Experimental[1] and predicted adsorption-desorption (top) and strain (bottom) isotherms for pure $CO_2$ at 254 K and $CH_4$ at 250 K.*

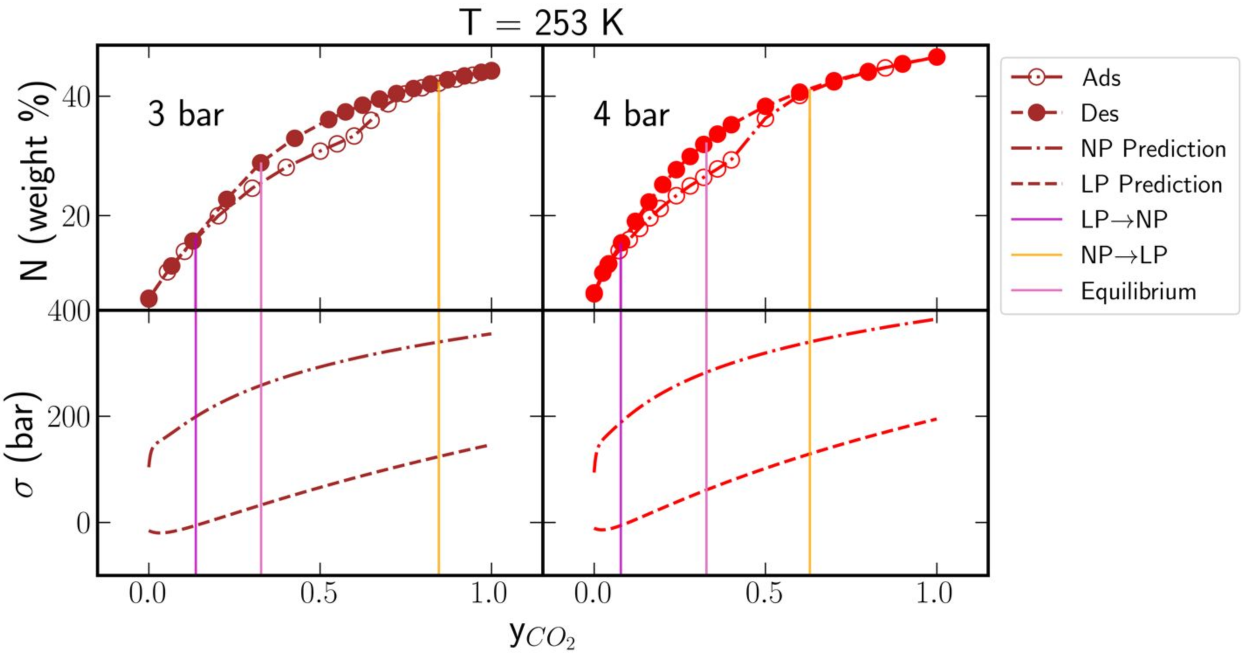


*Figure 3: Experimental adsorption[2] (top) and strain (bottom) isobar-isotherms at two pressures for a mixture of CH4 and CO2 at 253 K.*

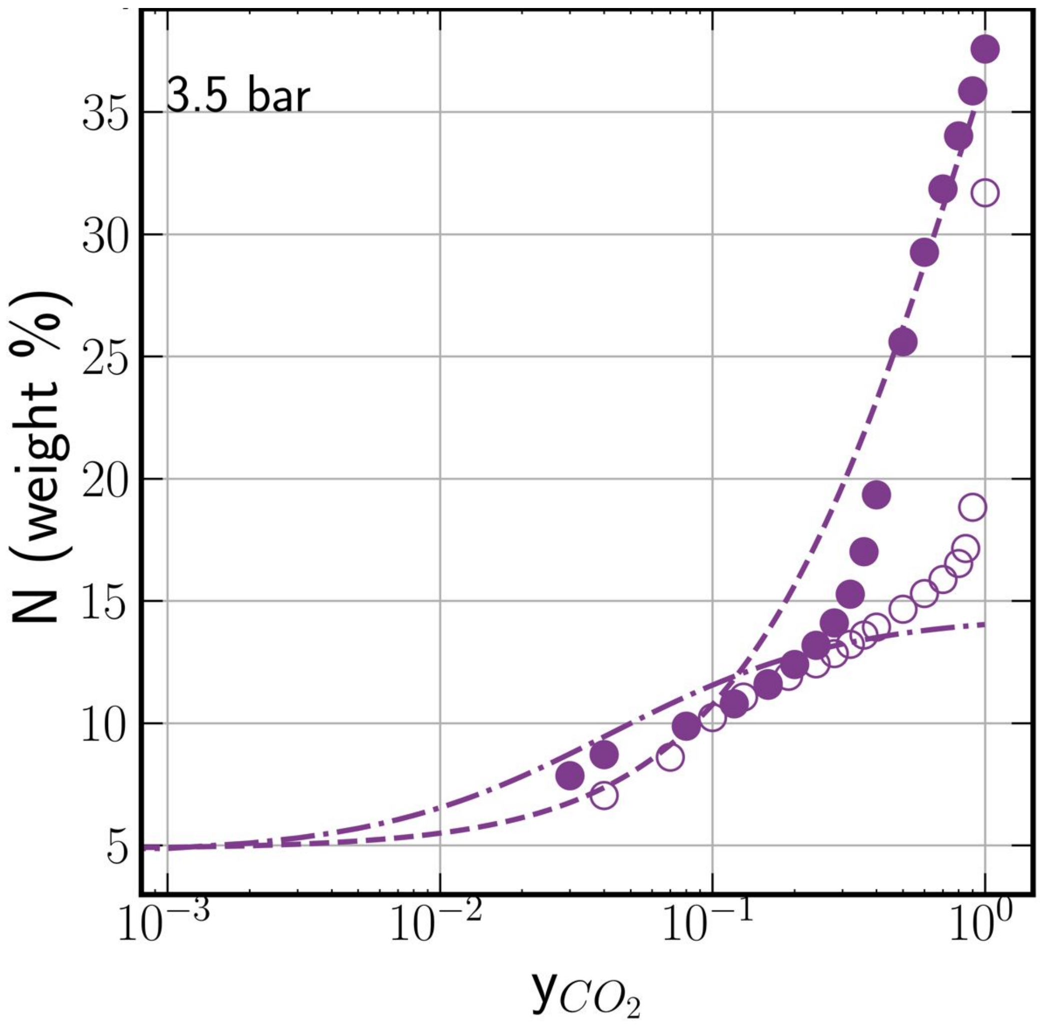


*Figure S4: The adsorption isobar-isotherms for p=3.5 bar from in Figure 3 of the main text presented in semi-logarithmic scale to show clearly the phase transitions*

1. Boutin, A., et al., *The behavior of flexible MIL-53 (Al) upon CH4 and CO2 adsorption.* J. Phys. Chem. C, 2010. **114**(50): p. 22237-22244.
2. Ortiz, A.U., et al., *Predicting Mixture Coadsorption in Soft Porous Crystals: Experimental and Theoretical Study of CO2/CH4 in MIL-53(Al).* Langmuir, 2012. **28**(1): p. 494-498.
